# How do researchers determine the difference to be detected in superiority trials? Results of a survey from a panel of researchers

**DOI:** 10.1186/s12874-016-0195-2

**Published:** 2016-07-29

**Authors:** Angèle Gayet-Ageron, Anne-Sophie Jannot, Thomas Agoritsas, Sandrine Rudaz, Christophe Combescure, Thomas Perneger

**Affiliations:** 1 Division of clinical-epidemiology, Department of health and community medicine, University of Geneva & University Hospitals of Geneva, Geneva, Switzerland; 2 Department of Medical Informatics and Public Health, Hôpital Européen Georges Pompidou, Assistance Publique Hôpitaux de Paris, Paris, France; 3 Department of Internal Medicine, Rehabilitation and Geriatrics, Geneva University Hospitals and Geneva Faculty of Medicine, Rue Gabrielle Perret-Gentil 4, 1211 Geneva 14, Switzerland

**Keywords:** Superiority trial, Randomized controlled trials, Difference to be detected, Minimal clinically important difference, Odds ratio, Primary outcome

## Abstract

**Background:**

There is currently no guidance for selecting a specific difference to be detected in a superiority trial. We explored 3 factors that in our opinion should influence the difference to be detected (type of outcome, patient age group, and presence of treatment side-effects), and 3 that should not (baseline level of risk, logistical difficulties, and cost of treatment).

**Methods:**

We conducted an experimental survey using a factorial design among 380 corresponding authors of randomized controlled trials indexed in Medline. Two hypothetical vignettes were submitted to participants: one described a trial of a new analgesic in mild trauma injuries, the other described a trial of a new chemotherapy among cancer patients. The first vignette tested the baseline level of risk, patient age-group, patient recruitment difficulties, and treatment side-effects. The second tested the baseline level of risk, patient age-group, type of outcome, and cost of treatment. The respondents were asked to select the smallest gain of effectiveness that should be detected by the trial.

**Results:**

In vignette 1, respondents selected a median difference to be detected corresponding to an improvement of 7.0 % in pain control with the new treatment. In vignette 2, they selected a median difference to be detected corresponding to a reduction of 5.0 % in mortality or cancer recurrence with the new chemotherapy. In both vignettes, the difference to be detected decreased significantly with the baseline risk. The other factor influencing difference to be detected was the age group, but the impact of this factor was smaller. Cost, side-effects, outcome severity, or mention of logistical difficulties did not significantly impact the difference to be detected selected by participants.

**Conclusions:**

Three of the anticipated effects conformed to our expectations (the effect of patient age, and absence of effect of the cost of treatment and of patient recruitment difficulties) and the other three did not. These findings can guide future research in determining differences to be detected in trials that can translate to meaningful clinical decision-making.

**Electronic supplementary material:**

The online version of this article (doi:10.1186/s12874-016-0195-2) contains supplementary material, which is available to authorized users.

## Background

When planning a randomized controlled trial that aims to demonstrate the superiority of a new treatment, researchers must decide on the magnitude of the difference in the outcome variable that they want to be able to detect between the two study arms. The difference should be large enough to lead to a change in clinical practice if the trial result is positive [1], but there are few formal recommendations on how to select a meaningful difference [2–6]. A reasonable starting point is the minimal clinically important difference, the smallest improvement in treatment efficacy that would matter to an individual patient [7, 8], but on occasion researchers may select a target difference that is smaller or larger than the minimal clinically important difference [9]. Because the difference to be detected reflects the investigators’ *a priori* opinion about a clinically important difference, referring to the original research hypothesis can help interpreting whether the observed difference is clinically significant or not [1, 4].

In our opinion, several factors can influence the choice of the difference to be detected in trials. Among the potentially legitimate ones is the type of primary outcome [10, 11]. When the primary outcome is mortality, even a small improvement would be considered important, whereas when the outcome is a less threatening event, a larger difference may be required [10]. Second, the anticipated harm, toxicity and burden of the new treatment could also play a role [6, 11, 12]. If the new treatment is expected to be safe, and convenient, even a slight improvement in the patient condition could be deemed important [8, 11–13]. Conversely, if the new treatment had side effects or was burdensome, investigators might aim for larger expected differences. Third, the age-group of the target population may also influence the choice of the difference to be detected. A smaller difference in the key outcome might be required in children than in older patients, because the longer life-expectancy could amplify the effect of a treatment. A smaller difference in the key outcome, such as death or disease progression may also be more desirable in children than in older patients.

In contrast, some factors should not influence the choice of the difference to be detected, such as the cost of the new treatment or the existence of logistical constraints in the implementation of the study. Indeed, these factors have nothing to do with clinical importance. Similarly, the baseline risk should in principle not influence the choice of the difference to be detected if it is expressed on an absolute risk scale, because the avoidance of an undesirable event has the same clinical relevance regardless of baseline risk (e.g., one life saved is saved regardless of baseline mortality) [14].

In this study, we explored if the candidate factors cited above influence the choice of specific differences to be detected by clinical researchers. We conducted a vignette-based experimental survey among a self-selected sample of corresponding authors who have published the results of a randomized controlled trial between 2010 and 2012, and were thus familiar with the design of clinical trials.

## Methods

### Study design and participants

We conducted a vignette-based study that included an experimental randomized design which was previoulsy published [14]. We selected a random sample from a list of corresponding authors who have published the results of a randomized controlled trial recorded in Medline between January 1, 2010 and December 31, 2012, and invited them by email to answer an online survey. The invitation message informed them that their participation was voluntary and that the return of the questionnaire signified consent to participate.

To identify relevant email addresses, we performed a Medline search with the MeSH terms “randomized controlled trial” OR (“randomized” AND “controlled” AND “trial”), and retrieved the corresponding author’s email address from the abstract or from the full article. We excluded corresponding authors of ancillary analyses of previously published studies, review articles, or nonhuman research. Because it carried minimal risk, the project was exempted from formal review by the institutional research ethics committee.

### Questionnaire and clinical vignettes

We created an electronic survey on Limesurvey (Limesurvey Project, Hamburg, Germany). The first section of the questionnaire assessed the respondent’s experience in clinical research. The second section included four clinical vignettes presented in a fixed order in all versions of the questionnaire. Two vignettes involved noninferiority trials, and the results have been previously published [14]. This study focuses on two vignettes presenting a superiority trial. The first superiority vignette described a hypothetical trial assessing the effect of a new analgesic *vs*. the standard of care in patients with mild trauma injuries (Additional file 1). The second vignette described a trial comparing a new adjuvant chemotherapy *vs*. standard therapy to reduce mortality or cancer recurrence among adults with an unnamed cancer (Additional file 2). The survey ended with questions on socio-demographic characteristics, education and training in research methods, and current position [14].

Each clinical vignette tested four binary factors in a factorial design. This yielded 16 versions of the survey which were randomly attributed to the email addresses previously retrieved (the addresses were sorted in random order and each 16^th^ of the list was directed to a different version of the survey). Participants were allowed to opt out and decline further invitations. A total of three reminders were sent to nonrespondents.

### Experimental factors and outcome variables

In both vignettes, low *vs*. high baseline risk (90 *vs*. 50 % of controlled pain in vignette 1 and 10 % *vs*. 60 % of mortality or cancer recurrence in vignette 2) and age-groups (adults *vs*. children populations in vignette 1 and less than 50 years *vs*. above 75 years in vignette 2) were tested. In addition, in vignette 1, the type of side effects (minor digestive *vs*. severe allergy) and the mention of difficulties to recruit patients for the trial (*vs*. no mention) were tested. In vignette 2, the severity of the primary outcome (mortality *vs*. cancer recurrence) and the mention of a high cost (*vs*. no mention), were also tested. All experimental factors were defined *a priori* based on a previous study from our group [10] and from review of the literature [8, 11–13].

At the end of each clinical vignette, we asked the respondent to select the proportion of the outcome expected in the new treatment group. Then we secondly computed the smallest gain of effectiveness (primary outcome) that would lead them to conclude that the new treatment was superior to the comparator. Effectiveness was expressed as the difference in the proportions of patients with the outcome of interest between the new treatment and the comparator (in vignette 1, gain in pain control; in vignette 2, reduction of mortality or cancer recurrence). A list of response options was proposed after each vignette, as well as an open field where the respondent could submit any other value (Table 1). At the suggestion of a reviewer, we also calculated odds ratios to assess the treatment effect selected by respondents.

**Table 1:** Response options proposed to participants in the two clinical vignettes depending on baseline risk used

	Proportion of pain control in the experimental group when baseline risk was:	
Vignette 1	Low (90 %)	High (50 %)
Trial of a new analgesic to control pain in mild trauma injuries.	90.5 91.0 92.0 95.0 97.0 100.0 Open field	51.0 55.0 60.0 70.0 80.0 90.0 Open field
	Proportion of mortality/cancer recurrence in the experimental group when baseline risk was:	
Vignette 2	Low (10 %)	High (60 %)
Trial of a new adjuvant chemotherapy following primary surgery for an unnamed cancer in adults.	9.9 9.5 9.0 8.0 5.0 0.0 Open field	59.9 59.5 58.0 55.0 50.0 40.0 30.0 Open field

### Sample size estimation

We sought to detect a standardized effect size of 0.3 for each factor independently, leading to a total number of about 470 participants (with a type 1 error of 5 % and type 2 error of 10 %). Anticipating a low response rate (around 25 %), we estimated that 2’000 emails should be sent in order to obtain an adequate number of participants.

### Statistical analysis

We described the characteristics of respondents who completed at least one of the two vignettes. Continuous variables were described by their mean and standard deviation (SD), median and range; categorical variables by their frequency and proportion by category.

The two vignettes were analyzed separately. Because of the factorial design, we directly constructed a multivariable linear regression model with the four experimental factors as the independent variables. Because the versions of the vignette were randomly attributed to participants, we did not adjust for the researchers’ characteristics. We did not predefine interactions to be tested. We obtained the estimated marginal means of the difference to be detected with their 95 % confidence intervals (95 % CI) for each experimental factor from the multivariable models and the associated *P*-value for each category of the factors. We verified graphically if the residuals of the model were normally distributed.

Finally, in order to estimate the odds ratio selected by respondents by experimental factor tested, we constructed two additional multivariable linear regression models with the four experimental factors as the independent variables (one per vignette) and as dependent variable the *ln*(odds ratio) computed from the risk in the control group and the respondent’s answer. We obtained the estimated marginal mean odds ratios with their 95 % confidence intervals (95 % CI) by the exponents of the estimates and confidence bounds.

All analyses were performed using STATA version intercooled 14 (STATA Corp., College Station, Texas, USA). Statistical significance was defined as *P* < 0.05 (two-sided).

## Results

### Sample characteristics

We first extracted 2'000 email addresses from abstracts published in 2010, then extended the search to December 31, 2012 due to the lower than expected response rate. In the end 6’374 invitation emails were sent out, 419 (6.6 %) researchers completed the online questionnaire and 380 (90.7 % of 419) answered at least one of the two superiority vignettes. Because recruitment was difficult and labor-intensive, we stopped enrollment before the target of 470 participants was achieved.

Respondents were on average 48.2 years old, more than two thirds were men, and most were from a European or North American country (75.9 %) (Table 2). The majority had participated in fewer than 10 randomized controlled trials in the past (64.2 %) and 47.4 % had participated in trials funded by the industry. They worked in diverse medical areas; 17.4 % worked in pain control and 11.1 % in oncology, the two areas illustrated in the vignettes. Less than a half had a degree in quantitative research methods. More than half of the participants reported being familiar with sample size estimation. Less than half of the participants considered themselves to be experts in determining the difference to be detected.

**Table 2:** Participants’ characteristics

Variables (number of available data)	Respondents (*n* = 380^a^)
Male gender, n (%) (*n* = 370)	258 (69.7)
Mean age (±standard deviation [SD], *n* = 370)	48.2 (±10.2)
Country of residence, n (%) (*n* = 370)	
North America South America Africa Asia Europe Oceania (Australia, New Zealand)	109 (29.4) 21 (5.7) 10 (2.7) 38 (10.3) 172 (46.5) 20 (5.4)
Mean number of past randomized controlled trial (RCT), n (%) (*n* = 380)	
1–5 6–10 11–20 >20	161 (42.4) 83 (21.8) 50 (13.2) 86 (22.6)
Proportion of RCT funded by industry, n (%) (*n* = 380)	
None 1–25 % 26–50 % > = 51 %	180 (47.4) 87 (22.9) 44 (11.6) 69 (18.2)
Work in pediatrics, n (%) (*n* = 380)	59 (15.3)
Work in pain control, n (%) (*n* = 380)	66 (17.4)
Work in oncology, n (%) (*n* = 380)	42 (11.1)
Training in epidemiology, n (%) (*n* = 380)	44 (11.6)
Training in Medicine, n (%) (*n* = 380)	252 (66.3)
Training as a nurse, n (%) (*n* = 380)	16 (4.2)
Training in psychology, n (%) (*n* = 380)	32 (8.4)
Training in statistics, n (%) (*n* = 380)	15 (3.9)
Have received a degree in quantitative research methods, n (%) (*n* = 370)	158 (42.7)
Have received formal training in “Good Clinical Practices”, n (%) (*n* = 370)	269 (72.7)
Member of an Ethics committee for research, n (%) (*n* = 370)	114 (30.8)
Mean percent of work time in health care (±SD, *n* = 370)	36.9 (±32.8)
Mean percent of work time in research (±SD, *n* = 369)	46.7 (±30.6)
Familiarity with the definition of the research question, n (%) (*n* = 380)	
Not familiar Familiar Expert	2 (0.5) 138 (36.3) 240 (63.2)
Familiarity with the choice of the primary outcome, n (%) (*n* = 380)	
Not familiar Familiar Expert	4 (1.0) 124 (32.6) 252 (66.3)
Familiarity with the estimation of the sample size, n (%) (*n* = 380)	
Not familiar Familiar Expert	25 (6.6) 229 (60.3) 126 (33.2)
Familiarity with the choice of the difference to be detected, n (%) (*n* = 380)	
Not familiar Familiar Expert	20 (5.3) 206 (54.2) 154 (40.5)
Familiarity with the selection of statistical methods, n (%) (*n* = 380)	
Not familiar Familiar Expert	40 (10.5) 237 (62.4) 103 (27.1)

^a^% calculated on available data, missing were excluded

### Factors explaining the choice of the difference to be detected

Among the 380 participants having answered at least one of the two superiority vignettes, 370 completed the first vignette and 353 the second vignette (343 completed both). The participants’ characteristics were evenly distributed between the 16 groups randomized to different versions of the vignettes (data not shown).

**Fig. 1:**
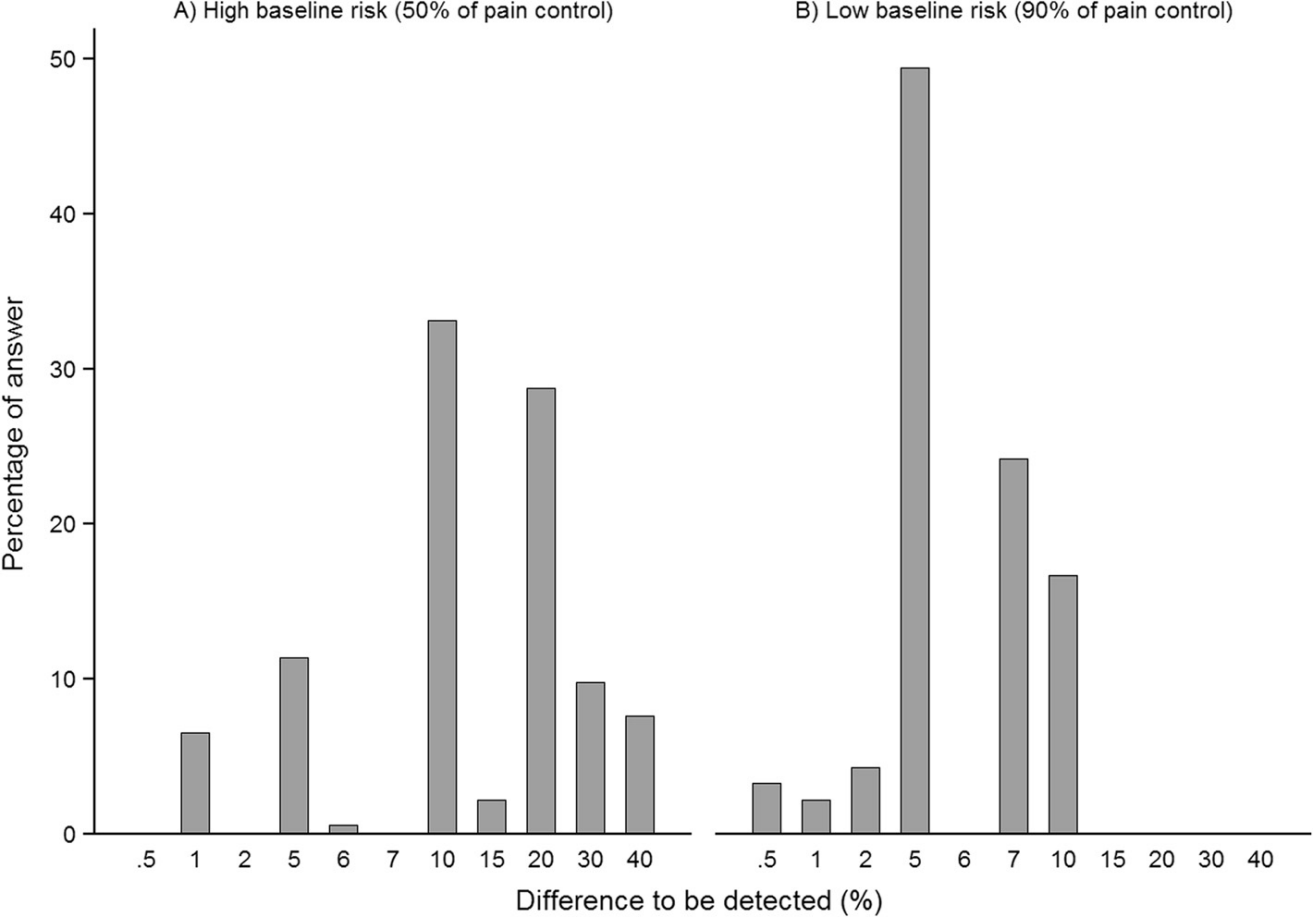
Distribution of the differences to be detected for controlled pain between the new treatment and its comparator selected by respondents in vignette 1 when: **a** the pain was controlled in 50 % of patients with reference treatment (high baseline risk) (*n* = 184), **b** the pain was controlled in 90 % of patients with reference treatment (low baseline risk) (*n* = 186). The difference between the percentage selected by a respondent and the baseline risk is the difference to be detected

Most respondents used the closed-format response scales; only 7 (5 in the first vignette) used the open field. In the first vignette, the mean proportion of controlled pain with new treatment selected by participants was ~96.0 % (SD 2.4; median 95.0 %; range 90.5 %–100.0 %) of controlled pain when baseline risk was 90.0 % (*n* = 186) and 66.0 % (SD 10.5; median 60.0 %; range 51.0 %-90.0 %) when baseline risk was 50.0 % (*n* = 184). These values corresponded to a mean gain of effectiveness or difference to be detected of +6.0 % (SD 2.4; median +5.0 %; range +0.5 % to +10.0 %) when baseline risk was low and +16.0 % (SD 10.4; median +10.0 %; range +1.0 % to +40.0 %) when baseline risk was high. Figure 1 presents the percentages of answer in each corresponding groups of gain of effectiveness depending on the baseline risk.

**Fig. 2:**
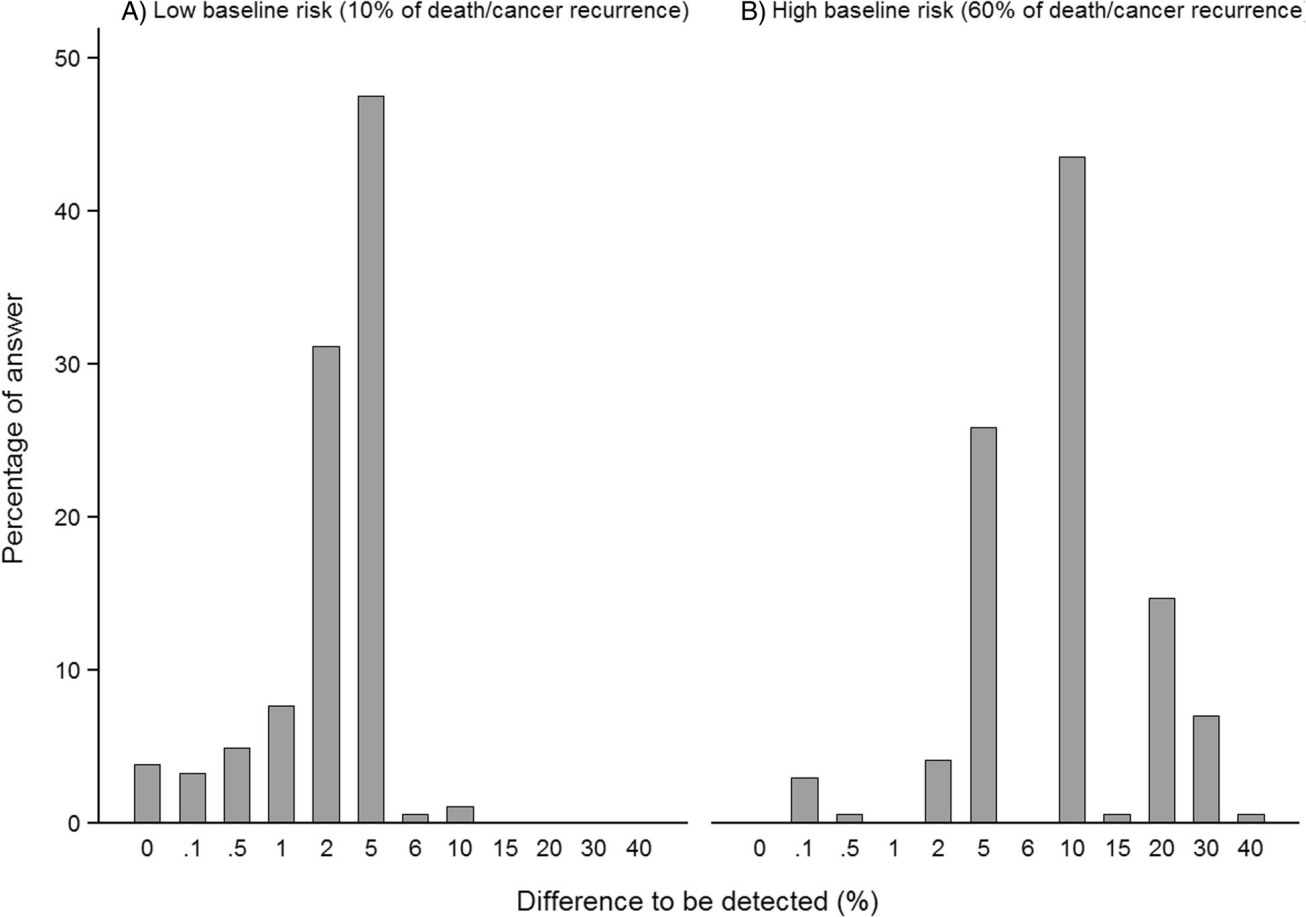
Distribution of the differences to be detected for death or cancer recurrence between the new treatment and its comparator selected by respondents in vignette 2 when: **a** the risk of death of cancer recurrence was 10 % in patients with reference treatment (low baseline risk) (*n* = 183), **b** the risk of death of cancer recurrence was 60 % in patients with reference treatment (high baseline risk) (*n* = 170). The difference between the percentage selected by a respondent and the baseline risk is the difference to be detected

In the second vignette, the mean proportion of death or cancer recurrence with new treatment selected by respondents was 6.8 % (SD 2.0; median 8.0 %; range 0.0 %–10.0 %) when baseline risk was 10.0 % (*n* = 183) and seven participants selected the 10.0 % answer in this group. When baseline risk was 60.0 %, participants selected a mean 48.9 % (SD 7.7; median 50.0 %; range 20.0 %–59.9 %) (*n* = 170). These values corresponded to a mean difference to be detected of −3.2 % (SD 2.0; median −2.0 %; range 0.0 % to −10.0 %) when baseline risk was low and −11.1 % (SD 7.7; median −10.0 %; range −0.1 % to −40.0 %) when baseline risk was high. Figure 2 presents the percentages of answer in each corresponding groups of difference to be detected depending on the baseline risk.

We secondly estimated the mean differences to be detected selected by respondents after adjustment for all experimental factors. In multivariable analyses, for both clinical vignettes, baseline risk was significantly associated with the difference to be detected (Tables 3 and 4). It was significantly larger by +10 % on average when uncontrolled pain was 50 % at baseline compared to 10 % (Table 3). It was significantly larger by +8 % on average when the mortality or recurrence rate at baseline was 60 % compared to 10 % (Table 4). In first vignette, we did not show that the difference to be detected was significantly associated with the age-group nor with the grade of the treatment side-effects. In second vignette, the difference to be detected was significantly larger, but by only 1.7 % on average, for younger cancer patients compared with older patients. We did not find a statistically significant association between the difference to be detected and the severity of the outcome assessed. Finally, we did not find that the difference to be detected was significantly associated with recruitment difficulties (Table 3) or with the treatment cost (Table 4).

**Table 3:** Experimental factors associated with the difference to be detected to control pain in mild trauma injuries presented as an absolute risk difference and as odds ratios

	Difference to be detected		Difference to be detected expressed as odds ratio	
Factors tested	Mean gain in pain control^a^, % (95 % CI)	*P*-value	Mean odds ratio (95 % CI)	*P*-value
Baseline risk		<0.001		0.017
Low risk (90 % of controlled pain) (*n* = 186) High risk (50 % of controlled pain) (*n* = 184)	6.0 (4.9–7.1) 16.0 (14.9–17.1)		2.3 (2.1–2.5) 2.0 (1.9–2.2)	
Study population		0.065		0.082
Adults (*n* = 188) Children (*n* = 182)	10.3 (9.2–11.3) 11.7 (10.6–12.8)		2.1 (1.9–2.2) 2.3 (2.1–2.4)	
Difficulties to recruit patients in the trial		0.111		0.029
No difficulty to recruit patients (*n* = 183) Difficulties to recruit patients (*n* = 187)	10.3 (9.3–11.4) 11.6 (10.5–12.7)		2.0 (1.9–2.2) 2.3 (2.1–2.5)	
Disadvantages of the new treatment		0.135		0.123
Risk of minor digestive side effects (*n* = 183) Risk of severe allergic reactions (*n* = 187)	10.4 (9.3–11.5) 11.6 (10.5–12.6)		2.1 (1.9–2.2) 2.2 (2.1–2.4)	

*95 % CI* 95 % confidence interval

^a^Marginal means from the multivariable linear regression model

**Table 4:** Experimental factors associated with difference to be detected regarding death or recurrence of an unnamed cancer presented as an absolute risk difference and as odds ratios

	Difference to be detected		Difference to be detected expressed as odds ratio	
Factors tested	Mean reduction in death or cancer recurrence^a^, % (95 % CI)	*P*-value	Mean odds ratio (95 % CI)	*P*-value
Baseline risk		<0.001		0.930
Low risk (10 % of mortality/recurrence) (*n* = 183) High risk (60 % of mortality/recurrence) (*n* = 170)	3.2 (2.4–4.0) 11.2 (10.3–12.0)	0.230	0.64 (0.61–0.67) 0.63 (0.60–0.66)	
Primary outcome				0.322
Mortality (*n* = 174) Recurrence rate (*n* = 179)	6.7 (5.9–7.5) 7.4 (6.6–8.2)	0.649	0.65 (0.62–0.68) 0.62 (0.60–0.65)	
Disadvantages of the new treatment				0.206
Higher cost mentioned (*n* = 180) Higher cost not mentioned (*n* = 173)	7.2 (6.4–8.0) 6.9 (6.1–7.7)	0.005	0.62 (0.59–0.65) 0.65 (0.62–0.68)	
Study population				0.031
Adults aged <50 years (*n* = 180) Adults aged >75 years (*n* = 173)	7.9 (7.1–8.7) 6.2 (5.4–7.0)		0.61 (0.59–0.64) 0.66 (0.63–0.69)	

*95 % CI* 95 % confidence interval

^a^Marginal means from the multivariable linear regression model

### Factors associated with the odds ratio for treatment effect

For the first vignette, the odds ratios of improved pain control computed from risks selected by the respondents were significantly stronger when the baseline risk was 90 % than when it was 50 %, which runs in the opposite direction compared to the analysis of risk differences (Table 3). Furthermore, the odds ratios were also higher in presence of recruitment difficulties; this effect was not apparent in the analysis of risk differences. The two other factors were not associated with odds ratio.

In second vignette (Table 4), the odds ratios of patient outcome (death or cancer recurrence) were similar for the two levels of baseline risk, unlike for the analysis of risk differences. The odds ratio was also associated with the patient age: a greater risk reduction was observed for younger cancer patients compared with older patients.

## Discussion

In this study, we attempted to identify factors that influence the difference to be detected in clinical trials as determined by experienced trialists. We tested three factors that in our opinion should influence the difference to be detected (severity of outcome, patient age group, and presence of side-effects in the experimental treatment), and three that should not (baseline level of risk, recruitment difficulties, and cost of treatment). Only two observed effects were in conformity with our expectations: we found that neither recruitment difficulties nor treatment cost had any effect on the difference to be detected. The other four results ran against our hypotheses: baseline risk was a strong determinant of the difference to be detected, the effect of age was opposite to our expectations, and the presence of side-effects in the experimental treatment and outcome severity did not influence the difference to be detected. Of note, these results appeared substantially different when the participants’ responses were expressed on a multiplicative scale, as odds ratios. The main change was that a baseline risk was no longer associated with the difference to be detected (in terms of odds ratios), or only weakly.

We found that participants in this study selected differences to be detected smaller than differences to be detected observed in real life [10]. With only two vignettes it is unclear if this disparity is meaningful. However, it is possible that researchers responded in earnest to this survey, whereas in real life they are compelled to choose larger differences that lead to smaller sample sizes [15]. Alternatively the use of abbreviated hypothetical vignettes resulted in bias, and a consideration of full study protocols would have produced different answers [16].

The most disturbing finding was that the difference to be detected was considerably larger (by 8–10 %) when the baseline risk was high (50 or 60 %) than when the baseline risk was low (10 %). From an ethical standpoint, we find this difficult to justify. An unfavorable event avoided – whether it is death, cancer recurrence, or persistence of uncontrolled pain – should have the same value to patients and to society, regardless of baseline risk. Nonetheless, when we used a multiplicative scale, baseline risk was no longer associated with the choice of the outcome proportion under new treatment. Odds ratios appeared to be independent of baseline risk: the difference was small (2.0 versus 2.3) in the first vignette and nil in the second vignette (0.64 versus 0.63). The most likely hypothesis is that the respondents have computed mentally a relative risk or odds ratio, such that the same absolute difference appeared more impressive when the baseline risk was low [17]. For instance, in the low risk group the proportion of controlled pain on standard treatment was 90 %, and the respondents selected on average 95 % for the experimental treatment, an odds ratio of 2.11. This is more impressive than the odds ratio of 1.50 obtained in the high risk group, where the proportions of controlled pain were 50 % and 60 %. Another possible explanation would be that the respondents were influenced by the response scales that were proposed, which were more spread out for high baseline risks than for low baseline risks. In other words, the observed difference could be due to ascertainment bias. However, the response scales only reflected reality – risk cannot be reduced by more than 10 points when the baseline risk of unfavorable outcome is 10 %, but can be reduced by much more when baseline risk is 50 %. We believe that the role of baseline risk in choosing the difference to be detected should be addressed by trialists and that an ethically acceptable solution to this issue is needed.

Another unexpected finding was that the participants appeared to take into consideration the age group when selecting the difference to be detected, but the effect was opposite to our expectations. The respondents selected larger differences in both vignettes for children and younger adults than for older adults, which suggests that smaller benefits are less justifiable among younger patients than among the old. This runs against the “fair innings” argument which would lead to the opposite [18]. One possible explanation is that clinical trials are generally conducted at the very early phase of a new drug development where adverse events are not well known. In that case, researchers may be reluctant to include children or younger adults compared to older adults unless the expected clinical benefit is important.

Outcome severity did not influence the difference to be detected in our survey. This negative result might be due to an insufficient contrast between the outcomes that we tested (mortality *vs*. cancer recurrence). Indeed, in a study based on published trial reports, the difference to be detected was significantly smaller for mortality than for other outcomes [10]. Because the latter study was observational, it did not control for other trial characteristics that might cause confounding, unlike this experimental study.

The severity of side effects of the new drug was expected to influence the difference to be detected but we only found a small difference that was not statistically significant. Balancing potential benefits, harms, and burden of treatment is central to clinicians’ and patients’ decision-making, and estimates of treatment efficacy can only be interpreted contextually, along with potential undesirable outcomes [19]. For example, in life-threatening situations, potential harm is often immaterial, whereas small or uncertain benefit can be outweighed by substantial established harm or burden [20, 21]. A plausible explanation to our findings is that respondents focused on setting a difference to be detected for the primary efficacy outcome of the superiority trial. Although focusing on the primary outcome is necessary for sample size calculation, a more comprehensive determination of harms and benefits could facilitate the translation of research findings into meaningful decision, as increasingly advocated by the GRADE working group [22].

Two negative results were in conformity with our hypotheses. The respondents were not influenced by anticipated difficulties in patient recruitment. This is an encouraging result; indeed, methodologists frequently report that some researchers negotiate an achievable sample size for their trial by revising upward the difference to be detected [15]. That this did not occur may reflect either the hypothetical nature of our study, or the fact that no feedback about the required sample size was given during the survey. The other reassuring result was the lack of effect of the cost of the new treatment. Thus researchers appear to have an attitude similar to that of clinicians [23]. Arguably efficacy trials should not concern themselves with the cost-effectiveness of the new treatment, especially as they are conducted early in the life-cycle of a drug or device, when treatment costs are at the apex.

Several limitations of our study deserve mention. First, this survey was addressed to researchers who have participated at least one randomized controlled trial. We supposed that the corresponding authors were involved at least in the planning and conduct of the published trial but we do not know their actual role in the determination of the difference to be detected when the trial was planned. Nonetheless, their self-perceived expertise in sample size estimation and in the selection of the difference to be detected was fairly high. Second, we obtained a smaller sample size than planned, but the study was sufficiently powered to reveal several relevant associations. The low participation rate also raises a concern about selection bias. However, the comparisons between respondent subgroups should be internally valid, since the allocation to versions of the vignettes was at random. Third, as with all vignette-based studies, it is uncertain if the observed results would apply equally in real life. In particular, the vignettes described two specific clinical areas that did not necessarily correspond to the clinical expertise of the respondents. This may have caused difficulties for some respondents in selecting an appropriate response. Fourth, participants were likely influenced by the proposed response options, which differred for the low risk and high risk versions of the *scenarii*. However, this reflects the reality: a low risk cannot be lowered as much as a high risk. If we had used the same response scale for the two situations, the “high risk” group would have been prevented from considering larger reductions in risk that were plausible in their situation, but that were impossible for the “low risk” group. We acknowledge however that our procedure made it impossible to distinguish a true preference for a larger (or smaller) risk difference from ascertainment bias due to the use of a wider (or narrower) response scale. An open “free-response” format would have avoided this problem. However, in a pre-test, we had compared the open “free reponse” format to a list of pre-defined response options, and respondents had more difficulty with the open format. Nonetheless, future studies should explore the influence of the mode of response on the resulting respondent opinions. Finally, we have explored only 6 factors that may influence the choice of a difference to detect in a trial, other factors may be considered, such as the prevalence of the disease (common *vs*. rare), a range of less severe outcomes (pain relief, quality of life, etc.), or the funding and sponsorship of the study (private *vs*. public).

## Conclusions

Understanding the researchers’ reasoning in selecting a difference to be detected in a randomized trial is important. Patients should be reassured that the sample size is justified by scientific and public health arguments and is not only based on the feasibility of the study [15, 24]. We also suggest that future qualitative studies should explore trialists’ reasoning in selecting the difference to be detected. In parallel, researchers involved in the design of clinical trials, as well as patient representatives, should engage in a debate regarding the ethical issues that arise in the selection of a difference to be detected in a trial. Developing strategies to determine this difference contextually, in light of established or potential harms of burden of treatment, is another important avenue for research. In the meantime, if a specific minimal clinically important difference exists in the literature, this may constitute a good starting point for selecting plausible values.

## Abbreviations

95 % CI, 95 % confidence interval; SD, standard deviation

## Additional files

Additional file 1: First version of the clinical vignette related on a new analgesic to control pain in mild trauma injuries with the four experimental factors tested. Description of first clinical vignette and list of response options. (DOCX 11 kb) Additional file 2: Second version of the clinical vignette related on patients presenting a non-named cancer with the four experimental factors tested. Description of second clinical vignette and list of response options. (DOCX 12 kb) Additional file 3: Study dataset. (XLS 262 kb)
