# Supplementary material for: How do researchers determine the difference to be detected in superiority trials? Results of a survey from a panel of researchers
Source: BMC Med Res Methodol. 2016 Jul 29;16:89. doi: 10.1186/s12874-016-0195-2 (PMC4966776; doi:10.1186/s12874-016-0195-2)
Supplement: Additional file 1: — First version of the clinical vignette related on a new analgesic to control pain in mild trauma injuries with the four experimental factors tested. Description of first clinical vignette and list of response options. (DOCX 11 kb) [file 12874_2016_195_MOESM1_ESM.docx]

**Additional file 1.** First version of the clinical vignette related on a new analgesic to control pain in mild trauma injuries with the four experimental factors tested (underlined).

A randomized controlled trial will test a new analgesic to control pain in trauma injuries. In adults, pain is usually controlled successfully in 50% of patients with standard treatment (Opidrug^TM^).

The trial will test a new treatment (Painkill^TM^) that is potentially more effective, but with more digestive side-effects (nausea, vomiting) than Opidrug^TM^. Despite the positive results in preliminary studies among adults, the investigators have major difficulties in convincing patients to participate in the trial. The purpose of the trial is to assess the superiority of the new drug Painkill^TM^ to control pain compared to Opidrug^TM^.

In designing the study, the investigators must determine the smallest difference with standard treatment that they would not want to miss. What is the smallest rate of pain relief with Painkill^TM^ that you would consider as substantially higher than the 50% rate with Opidrug^TM^?

| Please select your answer in the list |
| --- |
| a) 51% |
| b) 55% |
| c) 60% |
| d) 70% |
| e) 80% |
| f) 90% |
| h) Other, specify __________________ |
| i) I do not know |
