# Supplementary material for: How do researchers determine the difference to be detected in superiority trials? Results of a survey from a panel of researchers
Source: BMC Med Res Methodol. 2016 Jul 29;16:89. doi: 10.1186/s12874-016-0195-2 (PMC4966776; doi:10.1186/s12874-016-0195-2)
Supplement: Additional file 2: — Second version of the clinical vignette related on patients presenting a non-named cancer with the four experimental factors tested. Description of second clinical vignette and list of response options. (DOCX 12 kb) [file 12874_2016_195_MOESM2_ESM.docx]

**Additional file 2.** Second version of the clinical vignette related on patients presenting a non-named cancer with the four experimental factors tested (underlined).

Cancer remains one of the leading causes of death worldwide and accounted for 13% of all deaths in 2008 (WHO data). One specific cancer (voluntarily not named) is usually treated by adjuvant chemotherapy (OldchemoTM) following primary surgery and allows to achieve a 10% mortality rate within 12 months. A randomized controlled trial will test a new adjuvant chemotherapy (Newchemo^TM^) among adults with a life expectancy of between 10 to 20 years (normal).

The trial will test Newchemo^TM^, which is more expensive than standard therapy. The purpose of the trial is to assess the superiority of the new agent compared to Oldchemo^TM^ on the mortality rate at 1 year.

In designing the study, the investigators must determine the minimum difference with the current treatment that they would accept to consider Newchemo^TM^ as more efficient than Oldchemo^TM^ to control mortality. Which risk of death with Newchemo^TM^ would you choose to conclude that it is superior to the 10% risk with Oldchemo^TM^?

| Please select your answer in the list |
| --- |
| a) 9.9% |
| b) 9.5% |
| c) 9% |
| d) 8% |
| e) 5% |
| f) 0% |
| h) Other, specify __________________ |
| i) I do not know |
